# Evaluation of hidden hearing loss in normal-hearing firearm users

**DOI:** 10.3389/fnins.2022.1005148

**Published:** 2022-10-26

**Authors:** Sarah K. Grinn, Colleen G. Le Prell

**Affiliations:** ^1^Department of Communication Sciences and Disorders, Central Michigan University, Mount Pleasant, MI, United States; ^2^Department of Speech, Language, and Hearing, University of Texas at Dallas, Dallas, TX, United States

**Keywords:** synaptopathy, hidden hearing loss, noise induced hearing loss (NIHL), firearm noise, temporary threshold shift (TTS), speech-in-noise, Words in Noise (WIN), ABR Wave-I

## Abstract

Some noise exposures resulting in temporary threshold shift (TTS) result in cochlear synaptopathy. The purpose of this retrospective study was to evaluate a human population that might be at risk for noise-induced cochlear synaptopathy (i.e., “hidden hearing loss”). Participants were firearm users who were (1) at-risk for prior audiometric noise-induced threshold shifts, given their history of firearm use, (2) likely to have experienced complete threshold recovery if any prior TTS had occurred, based on this study’s normal-hearing inclusion criteria, and (3) not at-risk for significant age-related synaptopathic loss, based on this study’s young-adult inclusion criteria. 70 participants (age 18–25 yr) were enrolled, including 33 firearm users experimental (EXP), and 37 non-firearm users control (CNTRL). All participants were required to exhibit audiometric thresholds ≤20 dB HL bilaterally, from 0.25 to 8 kHz. The study was designed to test the hypothesis that EXP participants would exhibit a reduced cochlear nerve response compared to CNTRL participants, despite normal-hearing sensitivity in both groups. No statistically significant group differences in auditory performance were detected between the CNTRL and EXP participants on standard audiom to etry, extended high-frequency audiometry, Words-in-Noise performance, distortion product otoacoustic emission, middle ear muscle reflex, or auditory brainstem response. Importantly, 91% of EXP participants reported that they wore hearing protection either “all the time” or “almost all the time” while using firearms. The data suggest that consistent use of hearing protection during firearm use can effectively protect cochlear and neural measures of auditory function, including suprathreshold responses. The current results do not exclude the possibility that neural pathology may be evident in firearm users with less consistent hearing protection use. However, firearm users with less consistent hearing protection use are also more likely to exhibit threshold elevation, among other cochlear deficits, thereby confounding the isolation of any potentially selective neural deficits. Taken together, it seems most likely that firearm users who consistently and correctly use hearing protection will exhibit preserved measures of cochlear and neural function, while firearm users who inconsistently and incorrectly use hearing protection are most likely to exhibit cochlear injury, rather than evidence of selective neural injury in the absence of cochlear injury.

## Introduction

Noise-induced temporary threshold shift (TTS) in the absence of permanent threshold shift (PTS) can be accompanied by permanent damage to the synaptic connections between the cochlear inner hair cells (IHCs) and auditory nerve (AN) dendrites (cochlear synaptopathy) (see Kujawa and Liberman, 2009). A variety of data suggest that cochlear synaptopathy — inferred from evoked potential measurements — is correlated with hearing-in-noise difficulties in patients/participants who exhibit clinically normal-hearing sensitivity (thresholds 0.25–8 kHz ≤ 25 dB HL) (Grant et al., 2020, 2022; Mepani et al., 2021; Wu et al., 2021). Hazardous noise exposure is better known for compromising outer hair cell (OHC) integrity, and is associated with decreased distortion product otoacoustic emission (DPOAE) amplitude (Poling et al., 2022). Some data show correlations between DPOAE amplitude and hearing-in-noise function (Grinn et al., 2017; Parker, 2020). In addition to the studies noted above, a number of studies have failed to detect statistically significant relationships between either neural evoked potential amplitude or DPOAE amplitude with hearing-in-noise performance, raising questions about the sensitivity of the evoked potential metrics and other clinical tools, and the accuracy of noise report data (for review see Bramhall et al., 2019; Le Prell, 2019; Bramhall, 2021). Nevertheless, hearing-in-noise difficulty is a significant clinical issue that remains of high interest. Out of approximately 100,000 patient records reviewed at one clinic, 10% of the patients were seen for complaints of hearing-in-noise difficulty, despite their clinically normal audiometric test results (Parthasarathy et al., 2020).

Studies enrolling human participants in order to probe potential evidence of cochlear synaptopathy have followed several distinct approaches. The first of these approaches is enrollment of discrete groups differentiated by their history of a particular type of noise exposure (Bramhall et al., 2017; Grose et al., 2017; Bal and Derinsu, 2021; Megha et al., 2021; Nam et al., 2021; Suresh and Krishnan, 2021). A second approach is the enrollment of diverse participants with varied amounts of lifetime noise exposures, with lifetime exposure at least roughly quantified using survey tools (Prendergast et al., 2017a,b; Valderrama et al., 2018; Marmel et al., 2020). A third approach is the enrollment of normal-hearing young-adults with varied noise exposure histories and at least rough quantification of noise exposure in the past year, quantified using survey tools (Stamper and Johnson, 2015a,b; Fulbright et al., 2017; Grinn et al., 2017; Spankovich et al., 2017; Ridley et al., 2018). Finally, a prospective monitoring approach has been employed in a small number of studies, with baseline function measured and compared to data collected after a known noise exposure (Grinn et al., 2017; Wang et al., 2021). Results across diverse populations and methodological approaches have been mixed (for reviews, see Bramhall et al., 2019; Chen et al., 2019; Le Prell, 2019; Ripley et al., 2022), driving sustained interest in the identification of a damage-risk relationship for noise-induced cochlear synaptopathy in humans.

Within the data described in the human literature, one source of uncertainty is reliance on indirect electrophysiological measures, as cochlear synaptopathy cannot be directly measured *in vivo.* Additionally, there are significant uncertainties surrounding the precision of historical noise exposure estimates generated via survey data, which are a consequence of both errors in recall and lack of individual sound exposure level information. Therefore, more recent approaches have included the exploration of hearing-in-noise function (performance) for possible associations with measures of OHC function (typically assessed using DPOAEs) and/or measures of neural function using evoked potentials, such as the auditory brainstem response (ABR), acoustic reflex threshold/middle ear muscle reflex (MEMR), or envelope following response (EFR) (Grant et al., 2020; Mepani et al., 2020, 2021; Parker, 2020; Shehorn et al., 2020; Bramhall et al., 2022). At present, the audiogram remains the gold standard clinical tool in audiology (for recent review see Le Prell et al., 2022), but there is universal agreement that clinical dysfunction can be “hidden” beyond a normal audiogram (i.e., “hidden hearing loss”; see Schaette and McAlpine, 2011).

Although noise-induced cochlear synaptopathy has been widely documented in animal models, it is now clear that vulnerability to this pathology varies across species, with non-human primates being significantly less vulnerable to selective neural injury than rodents (Valero et al., 2017). Synaptic repair initially did not appear to occur in mice (Kujawa and Liberman, 2009). However, more recent data from a different mouse strain revealed spontaneous synaptic recovery (Kauer et al., 2019). Separately, synaptic recovery has been demonstrated with relative consistency in studies using guinea pigs as subjects (Liu et al., 2012; Shi et al., 2013; Song et al., 2016; Hickman et al., 2021).

It is not known if noise-induced cochlear synaptopathy recovers in humans or in non-human primates as it does in guinea pig, or if synaptopathic injury will remain permanent, as is most often the case in the mouse model. Importantly, even within mouse models that have not shown synaptic recovery, it has become clear that (1) not every noise exposure that induces TTS will result in cochlear synaptopathy, and (2) synaptopathy cannot be predicted solely by the TTS measured at a specific frequency (Fernandez et al., 2015; Fernandez et al., 2020). Finally, it must be highlighted that synaptopathic injury in experimental rodents is routinely induced by a single, acute noise exposure. Such an injury is a largely artificial condition relative to human participants who, by contrast, have a lifetime of accumulated effects of noise, age, and toxin exposures (Wu et al., 2021). However, data from impulse noise paradigms may provide an opportunity to address questions of noise-induced cochlear synaptopathy risk relative to humans, given that impulse noise has been shown to induce cochlear synaptopathy in rodent models (Altschuler et al., 2019), and impulse noise from firearm discharge could induce cochlear pathology from even a single, unprotected (or under-protected) exposure to firearm noise.

Indeed, one human population in which noise-induced cochlear synaptopathy has been interpreted as being both present and permanent is firearm users, as suggested in the work by Bramhall et al. (2017). Firearm users represent a specific human population of interest to evaluate possible risk for synaptopathic injury as high-intensity (peak level > 160 dB) impulsive sounds have been shown to induce TTS > 30 dB in humans (Fletcher and Loeb, 1967) and chinchillas (Henderson and Hamernik, 1986). According to nationally representative U.S. NHANES data, approximately 18% of U.S. children are exposed to firearm noise (Bhatt et al., 2020). Laffoon et al. (2019) reported hearing deficits at 2, 3, 4, 14, and 16 kHz in U.S. youth firearm users, as well as reduced DPOAE (pressurized) amplitude at 8 and 10 kHz; however, they did not measure sound-evoked neural potentials. With respect to adult cohorts, Bramhall et al. (2017) collected data from 64 participants with varying amounts of firearm experience and normal audiometric thresholds (≤ 20 dB HL 0.25–8 kHz) in at least one ear (32 participants qualified in both ears and 34 participants qualified in only one ear). The data from the better ear showed statistically significant differences in ABR Wave-I amplitude between those with and without firearm experience. Interestingly, middle latency response (MLR) amplitudes have been smaller in firearm users, corresponding to smaller Wave-I amplitudes, although late latency response (LLR) amplitudes were not reduced, suggesting central gain (Bramhall et al., 2020).

Extended high frequency (EHF) hearing is often compromised in participant populations in which cochlear synaptopathy has been inferred based on neural potentials (for recent review, see Lough and Plack, 2022). Although neural potentials were not measured, hunters in Cyprus were reported to have both conventional and EHF hearing deficits that preceded complaints of hearing difficulty (Tinazli and Tinazli, 2022). Engdahl and Aarhus (2022) did not test EHF frequencies, but reported hearing deficits at 3, 4, and 6 kHz in adult recreational firearm users participating in the Trondelag Health (HUNT) study in Norway. Thus, PTS is a common finding in studies that recruit adult firearm users (for review, see Meinke et al., 2017). DPOAE deficits, EHF hearing deficits, and deficits in sound-evoked neural potentials are often reported in studies of adult firearm users. Other studies have investigated TTS and/or PTS changes after firearm use using prospective designs (rather than the retrospective, cross-sectional study designs noted above) in order to measure acute changes in hearing, showing both DPOAE and audiogram deficits following firearm noise exposure (for review see Sonstrom Malowski et al., 2022).

Given that firearm use is reliably associated with changes in hearing sensitivity, it may be the case that young-adult firearm users are at risk for cochlear synaptopathic injury (based on the data from Bramhall et al., 2017, 2020). It also may be the case that tests which are suggested to be sensitive to cochlear synaptopathy will reflect auditory deficits in firearm users who have not yet developed overt hearing loss (typically defined as greater than 20–25 dB HL thresholds from 0.25 to 8 kHz). ABR Wave-I amplitude is of particular interest, given its sensitivity in animal models and widespread use in human studies (for review see Bramhall et al., 2019). Another evoked response of significant interest is the acoustic reflex/MEMR, which has been sensitive to noise-induced cochlear synaptopathy in rodents (Valero et al., 2016, 2018). MEMR did not vary with noise exposure in Guest et al. (2019) or Causon et al. (2020), although it was reduced in participants with noise-induced tinnitus in Wojtczak et al. (2017). The differences in findings may reflect differences in participant populations, but there were also significant differences in MEMR measurement techniques across studies. MEMR has good test-reliability (Guest et al., 2019; Kamerer et al., 2019), but the relationships between MEMR and functional measures such as hearing-in-noise performance are dependent on the MEMR protocol used (see Mepani et al., 2020).

Hearing-in-noise difficulty is of significant interest, and questions remain regarding relationships between cochlear synaptopathy and hearing-in-noise performance (for reviews, see Le Prell, 2019; Henry, 2022). Hearing-in-noise deficits have been documented after TTS noise exposure in rats (Lobarinas et al., 2017) and gerbils (Monaghan et al., 2020) and after cochlear ouabain application (a Na + K ATP-ase inhibitor that selectively eliminates type I spiral ganglion neurons) in mice (Resnik and Polley, 2021), but deficits were not observed after kainic acid-induced loss of auditory nerve fibers in budgerigars (parakeets) (Henry and Abrams, 2021). Several studies failed to find relationships between hearing-in-noise performance and lifetime noise exposure in humans (Guest et al., 2018; Marmel et al., 2020). Questions about causality of hearing-in-noise deficits (due to OHC loss and/or synaptic pathology) remain, with increasing recognition of confounded functional deficit interpretation when the pathology is mixed, given that many audiological tools lack precision (Sheppard et al., 2020).

## Materials and methods

### Recruitment

This study was approved by the Institutional Review Board at The University of Texas at Dallas. Written informed consent was obtained from participants prior to study enrollment. Participants were recruited from the University of Texas at Dallas campus in Richardson, Texas, and the Callier Center for Communication Disorders in Dallas, Texas. A digital flyer including study information was also shared via undergraduate and graduate student social media networks. The digital flyer contained a website link to a Qualtrics survey (a licensed, online survey software) which automatically screened interested participants to determine if they met the inclusion criteria. All study procedures were performed using dedicated clinical research equipment located at the Callier Center for Communication Disorders in Dallas, TX. All study procedures were conducted by a licensed audiologist or graduate students in the Doctor of Audiology program. Participants were allowed to withdraw from the study at any time, and they were compensated for their laboratory visit.

### Inclusion criteria

Seventy total participants were enrolled in this study. All participants were required to meet inclusion criteria including 18–25 years of age, normal otoscopic examination (no abnormalities, abrasions, or excess cerumen, defined as greater than 10% occluded view of the tympanic membrane), normal tympanometric examination (Type A, 226 Hz Tone), and bilaterally normal-hearing sensitivity (defined as ≤ 20 dB HL at all frequencies 0.25–8 kHz).

Interested participants clicked the Qualtrics website link in the digital advertisement, which prompted them to first complete an automated screening survey for study eligibility, including a question about previous firearm use. In the automated screening survey, interested participants were asked to estimate the number of occasions on which they had used firearms in the past, with available survey answers including 0, 1, 2, 3, 4, or 5 + occasions of firearm use. Participants who reported “0 occasions of firearm use” were assigned to the Non-Firearm User Control (CNTRL) group (*n* = 37; 25 female, 12 male). Participants who reported “5 + occasions of firearm use” were assigned to the Firearm User Experimental (EXP) group (*n* = 33; 12 female, 21 male). Participants who reported 1, 2, 3, or 4 occasions of firearm use were excluded from the study. The advertisement for the study intentionally did not include any reference to firearm exposure criteria, such that participants would not be biased in their response about their firearm exposure history.

### Firearm use survey and hearing protection device attenuation

All participants completed a survey about their previous firearm exposure that was specifically developed for use in this study. The survey was administered via Qualtrics online survey tool. Participants in the Firearm User (EXP) group were asked to estimate the total number of occasions on which they had used a firearm in their life (not the number of firearm discharges, but rather the number of occasions of firearm use), and they were asked to report the type(s) of firearm(s) that they had used in the past. Type of firearm options included handguns, shotguns, bolt-action rifles, semi-automatic rifles, or “Other: please describe the firearm,” which two participants chose, and both wrote-in “fully automatic rifle.” EXP participants were also asked how often they wore hearing protection during their reported total occasions of firearm use, with options including “Never”; “Less than half the time,” “About half the time”; “Almost all the time”; and “All the time”.

Participants were shown two types of hearing protection devices: (1) an earplug style HPD (3M EAR Classic Earplugs; 29 dB NRR), and (2) an earmuff style HPD (3M H10A Peltor Optime; 30 dB NRR). EXP participants were asked to pick which of the two types of HPD best represented the style of HPD that they personally use during firearm use, while CNTRL participants were asked which HPD style best represented the type of HPD they would use during “noisey activities.” In the event that participants did not use HPDs, they were asked to select which HPD style they would most likely wear during a noisy activity. All participants underwent real-ear-attenuation-at-threshold (REAT) HPD attenuation measurement testing in a laboratory setting using the HPD that they selected (3M EAR Classic Earplugs or 3M H10A Peltor Optime Earmuffs). REAT was performed from 0.25 to 8 kHz. REAT attenuation was calculated as the difference between hearing thresholds measured in a free-field condition without hearing protection and an occluded audiogram collected with hearing protection inserted. Participants were not given instruction or education about the HPDs, and were asked to self-fit the HPD for the REAT test. Given that only 4% of the total participants reported using HPDs during non-firearm noisy activities, REAT data were only relevant for the EXP participants who wore HPDs (i.e., the 32/33 participants who reported using HPDs at least some percentage of the time during firearm use).

### National acoustics laboratories “Know Your Noise” survey

**FIGURE 1:**
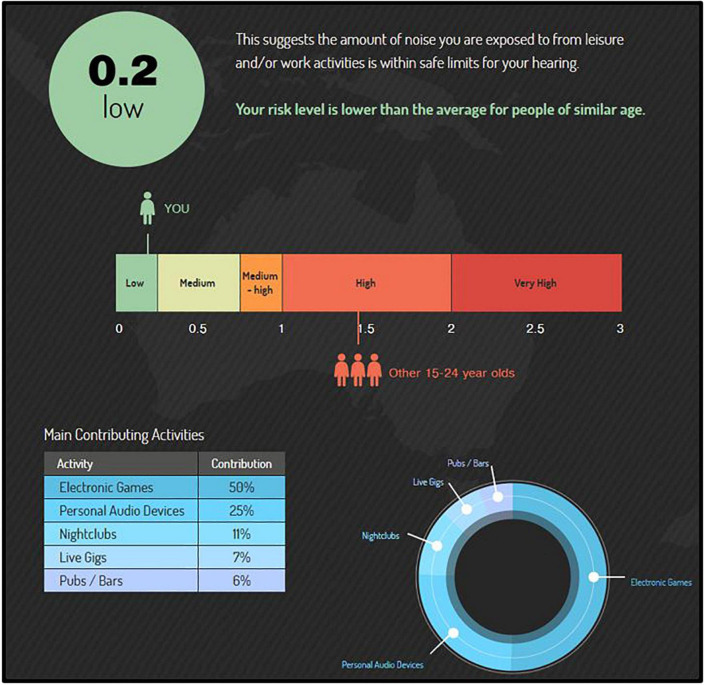
The “Noise Risk Calculator” (https://knowyournoise.nal.gov.au/noise-risk-calculator) is a free, online tool provided within the National Acoustics Laboratories (NAL) “Know Your Noise” campaign. The results shown here are from a participant with a score (score = 0.2) indicating lower risk than the daily NIOSH acceptable noise limit (score = 1.0), and lower risk on average than peers in their age group (average score = 1.4). The percentile breakdown of the participant’s most hazardous, typical activities is included in the bottom left corner.

All participants (CNTRL and EXP) completed the free, web-based “Noise Risk Calculator” survey, which uses the participant’s reported noise exposure history to predict their NIHL risk (see Gilliver and Beach, 2021). This 8--10 min online survey is available through a larger campaign (‘‘Know Your Noise’’) created by the National Acoustics Laboratories with funding from the Australian Government Department of Health.^1^ The survey user is asked various questions about common sources of hazardous noise in their current lifestyle, and assigns sound level estimates to these sources according to the NOISE database developed by the NAL (Beach et al., 2014). Upon completion, the survey user receives a noise exposure “score” and interpreted degree of their average daily NIHL risk according to their typical lifestyle (recreational and occupational) activities. A score of 0.0 to 0.25 indicates low risk, 0.25 to 0.75 indicates medium risk, 0.75 to 1.0 indicates medium-high risk, 1.0 to 2.0 indicates high risk, and a score greater than 2.0 indicates very high risk (for example, see Figure 1). Scores higher than 1.0 (“high risk”) indicate that the user’s noise exposure exceeds the recommended daily occupational noise exposure (a time-weighted average of 85 dB over 8 h) as recommended by the National Institute for Occupational Safety and Health (NIOSH), and scores over 2.0 (“very high risk”) indicate that the user’s noise exposure is more than double the NIOSH acceptable daily noise limit.

According to the survey’s database (see Beach et al., 2014; database updated as more user entries are made), a score of 1.4 is the norm (mean) for the age group 15 to 24 yr. Participant age range criterion in the current study was 18 to 25 yr. Upon survey completion, users are shown a percentile breakdown of the top sources of hazardous noise in their lifestyle that contribute to their risk of NIHL. Further, their overall NIHL risk score is compared to the NIOSH acceptable noise limit, as well an age-matched peer group. Importantly, while the survey asks about firearm noise exposure, it does not include firearm exposure into the Noise Risk Calculator score. As such, this survey provided an estimate of NIHL risk from non-firearm-related noise exposures for each participant (EXP and CNTRL). Lastly, this survey is freely conducted in the public network of the internet; every effort is made to assure the security of the website and accuracy of the updated data, but cannot be guaranteed.

### Otoscopy

Visual examination of the external ear and tympanic membrane were conducted to assure normal anatomy and no presence of debris. Normal otoscopic outcomes were defined as 90% visualization of the tympanic membrane (no more than 10% cerumen occlusion) with no apparent structural abnormalities.

### Tympanometry

Tympanometric measures were used to assess the functional status of the middle ear using a Grason Stadler Instruments TympStar Pro. Normal middle ear function was defined as Type A 226 Hz tone tympanograms bilaterally.

### Middle ear muscle reflex

Middle ear muscle reflex (MEMR) was measured in the right and left ears using Grason Stadler Instruments TympStar Pro equipment. Following Guest et al. (2019), MEMRs were elicited using 1 and 4 kHz tones. However, unlike Guest et al. (2019), MEMR threshold was not sought; instead, MEMR was recorded ipsilaterally for all participants at stimulus levels of 90 dB HL and 100 dB HL for frequencies 1 kHz and 4 kHz. MEMR recordings were repeated for each ear.

### Distortion product otoacoustic emissions

The 2f1-2 distortion product otoacoustic emission (DPOAE) was elicited with two simultaneously presented “primary” tones (f1 and f2) at an f2/f1 ratio of 1.22, with f2 frequencies of 1.5, 2, 3, 4, 5, 6, 8, 9, 10, 11, 12 kHz (f1: 65-dB SPL; f2: 55 dB SPL), with response measurements averaged over 4 sec. DPOAEs were measured with a handheld Grason Stadler Instruments Corti machine. Absolute amplitude of the DPOAE was measured for both the right and left ear independently. The noise floor level ranged from –18 to –20 dB SPL during recordings.

### Standard audiometry and extended high-frequency audiometry

Pure-tone air and bone conduction audiometry thresholds were obtained using the Grason Stadler Instruments Audiostar Pro. A modified Hughson-Westlake procedure and HDA 300 Senheiser headphones were used to obtain thresholds from 0.25 to 20 kHz, with sound levels decreased by 10-dB after each correct detection, and increased by 5-dB after each missed detection. All audiometric testing was conducted inside a sound-treated booth. Clinical significance of audiometry threshold differences was defined as 3 dB HL, based on reports of the just-meaningful-difference in the speech-to-noise ratio across various degrees of hearing sensitivity (McShefferty et al., 2015).

### Auditory brainstem response

An Interacoustics Eclipse EP25 was used to collect cochlear nerve response data. ABR data were collected following the procedures described by Grinn et al. (2017). A two-channel setup was used in the current study; however, recordings were essentially measured as a single-channel recording for the left and right ears. A two-channel setup was used to avoid potential human error when switching the electrode montage from right ear recordings to left ear recordings. Etymotic ER3-26A gold electrodes (tiptrodes) were placed inside the ear canals, and multipurpose cloth electrodes (Oaktree Products, Inc.) were positioned in the standard, adult diagnostic clinical configuration with non-inverting and ground electrodes stacked with spacing at midline high forehead (Fz). Skin surface for electrode placement was prepared with NuPrep gel.

A click stimulus and 4 kHz tone stimulus were independently presented at 90 dB nHL to all participants (Blackman, 5 cycles) in the right and left ears. Recording parameters included response filtering from 33 to 1500 Hz to increase the signal-to-noise ratio, alternating polarity, 11.7/s stimulus rate, and 500 sweeps of averaging, following Grinn et al. (2017) in which measurement repeatability was established. ABR Wave-I latency, Wave-I amplitude, Wave-V latency, and Wave-V amplitude were each marked by two trained reviewers. Waveform amplitude and latency were automatically calculated by Interacoustics Eclipse EP25 software subsequent to the reviewer’s placement of the waveform markings.

Two reviewers scored the waveforms, and were blinded to participant identifiers (e.g., firearm group, sex, age) and to each other’s scoring. All ABR waveforms were scored by two reviewers which were later compared against each other for the purpose of detecting significant differences, and were ultimately averaged. Reviewer 1 vs. Reviewer 2 comparison of waveform markers revealed several statistically significant differences in ABR measurements between reviewers (*p’s* < 0.05). However, the mean differences for the waveform markers differed by no more than hundredths of ms for waveform latency difference or hundredths of μV for waveform amplitude, indicating small but reproducible differences in the placement of the waveform marker at peaks or valleys. These small differences were not meaningful, as ABR Wave-I amplitude has a known coefficient of variation of 0.26 to 0.30 μV in humans (Guest et al., 2017). ABR measurements made by the two reviewers were therefore averaged for all group analyses.

### Words-in-Noise performance

Hearing-in-noise difficulty was assessed using the Words-in-Noise (WIN) test on the GSI Audiostar Pro following the procedures established by Wilson et al. (2003); for review, see (Wilson, 2011). A subset of the NU-6 words spoken by a female speaker is presented against multi-talker babble composed of 6 female voices. The babble is fixed at 80-dB SPL with target word level beginning at 104-dB SPL and decreasing in 4-dB steps from 104 to 80 dB SPL. 5 words are presented at each signal-to-babble (S/B) ratio, which decreases from 24 (easiest) to 0 (most difficult). The primary performance metric is the 50% correct point, or dB S/B threshold, calculated using the equation dB S/B = 26 – (0.8 × N), with N defined as the total number of correct words across all conditions (for review, see Wilson, 2011). Wilson and McArdle (2007) defined 3.5 dB-S/B as a clinically meaningful difference between scores, which corresponds to a difference of approximately 4 words out of the 35 words presented.

### Statistical analyses

Statistical analyses were performed using IBM SPSS Statistics software. Statistical significance was defined as *p* < 0.05 for all analyses. ABR measurement differences between Reviewer 1 and Reviewer 2 were analyzed using Paired *T*-Tests. With the exception of the MEMR measurement, all audiological measurements (tympanometry, audiometry, DPOAE, ABR) were assessed with a single run (i.e., single trial), as previous work using this protocol established no statistically or clinically significant differences between repeated trials (Grinn et al., 2017). MEMR was not included in our previous protocol; therefore, MEMR measurements were repeated and compared (Trial 1 vs. Trial 2) in the current protocol to assess repeatability. There were no statistically significant differences between Run 1 and Run 2 in either the left or right ear for MEMR in response to 1 kHz and 4 kHz tonal stimuli presented at 90 dB HL and 100 dB HL (*p*’s > 0.05), consistent with high test-retest reliability of the MEMR reported by Guest et al. (2019). Therefore, Run 1 and Run 2 of the MEMR were averaged for further analyses. The main and interaction effects of ear, sex, noise history score, and group (CNTRL and EXP) on auditory measures (audiometry, DPOAE, MEMR, ABR, WIN) were evaluated using linear mixed model analysis with repeated measures (right and left ear), with each auditory measure as the dependent variable in individual models. A Bonferroni correction was used in each model’s analyses of main effects. Correlations between noise history score and auditory measures was interpreted using Pearson’s correlation coefficient, with correlation strength interpreted following “Rule of Thumb for Interpreting the Size of a Correlation Coefficient” found in Hinkle et al. (2003).

## Results

### Trends in participant noise exposure

In the current study, 34% of participants (EXP and CNTRL groups) scored “high NIHL risk” or “very high NIHL risk,” with scores >1.0 and >2.0, respectively, on the “Noise Calculator” survey, which evaluates the frequency of non-firearm sources of hazardous noise exposure. For comparison, the “average Noise History Score” for 15 to 24 yr (males and females combined) is 1.4, which is interpreted as a level of NIHL risk that is “high” (see Figure 1, for example).

Only 3/70 participants (4%) reported using hearing protection devices (HPDs) during any of the (non-firearm) sources of hazardous noise that they reported (e.g., live music, power tools, video games). The very low consistency of HPD use reported in this study aligns closely with data from Eichwald et al. (2018), in which only 8% of adults (18 + yr) reported consistent HPD use during hazardously noisy recreational activities.

**FIGURE 2:**
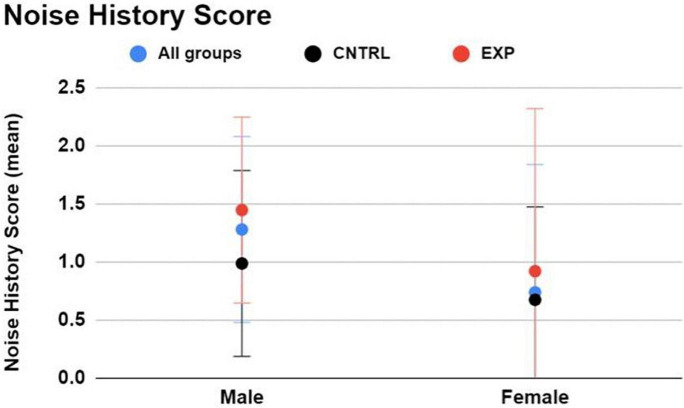
The Noise Risk Calculator (https://knowyournoise.nal.gov.au/noise-risk-calculator) computes a score to indicate the user’s NIHL risk level from everyday, noisy activities reported by the user. The estimation excludes risk of firearm use from the formula. Between males and females, males exhibited a higher Noise History Score than females. Between firearm groups, EXP participants exhibited a higher Noise History Score than CNTRL participants. Figure shows mean and one standard deviation, with higher NIHL risk indicated by a higher score.

**Between firearm groups (regardless of sex)**, and keeping in mind that this survey *does not* incorporate history of firearm use into the NIHL risk estimate, EXP participants exhibited higher Noise History Scores than CNTRL participants (average score = 1.3 ± 1.1 and 0.8 ± 0.8, respectively), indicating that the EXP group, on average, is more noise-exposed than the CNTRL group to non-firearm sources of hazardous noise (Figure 2).

**Between males and females (regardless of firearm group),** males exhibited a higher Noise History Score than females (average score = 1.3 ± 1.1 and 0.7 ± 0.8, respectively) (Figure 2).

**FIGURE 3:**
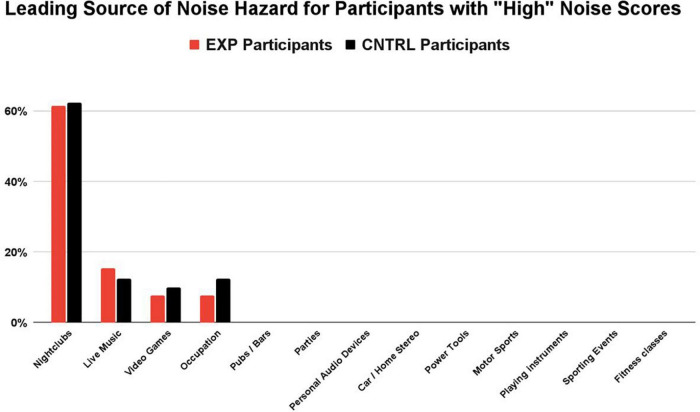
For participants with “high” noise scores (>1.0), nightclubbing was the largest contribution of noise hazard in both groups (62% EXP, 63% CNTRL), followed by attendance at live music gigs (15% EXP, 16% CNTRL). Noise sources with the highest contribution to the overall score were determined by their sound level and the participant’s frequency of exposure. While other sources of hazardous noise may have contributed to the overall noise score (e.g., pubs/bars, parties, personal audio devices), they were never the primary noise hazard contribution in those participants with “high noise” scores.

The Noise Calculator survey concludes with a percentage breakdown of each sound source’s contribution (i.e., percent contribution) to the overall noise score. For those participants with noise scores > 1.0 (the “High NIHL Score” subgroup), a distribution of the *primary noisy activity* (i.e., the exposure with the greatest contribution to their overall noise score, based on sound level and frequency of exposure) is shown for the EXP and CNTRL groups in Figure 3. The groups share the same four leading contributors to dangerously high noise scores: nightclubs, live music, video games, and occupation. No details were obtained about the occupation job titles. In summary, the most common, primary (non-firearm) source of auditory threat to both the EXP group and the CNTRL group was amplified music venues (nightclubs and live gigs). Nightclubbing was the largest contribution of noise hazard in both groups (62% EXP, 63% CNTRL), followed by attendance at live music gigs (15% EXP, 16% CNTRL). Hazardous noise sources such as pubs/bars, parties, personal audio devices, car/home stereo, power tools, motor sports, playing instruments, sporting events, and fitness classes — while contributing some amount of noise hazard in some participants — were never the primary noise hazard contribution in those participants with high noise scores.

### Firearm use survey results

The EXP group was limited to participants who had used a firearm on 5 + occasions (i.e., more than five individual occasions on which they had used a firearm, regardless of the number of times they discharged the firearm on each occasion) in their lifetime. The number of firearm use occasions (self-reported estimate) in the EXP group ranged from 5 to 200 occasions in their lifetime (mean = 28 ± 50). Four participant outliers reported occasion totals of 50 (*n* = 1), 100 (*n* = 1), and 200 (*n* = 2). Excluding the outliers, the number of estimated firearm use occasions ranged from 5 to 35 (mean = 11 ± 9). Participants were asked to report the type(s) of firearm(s) they had used in the past. Participants reported using handguns (*n* = 25), shotguns (*n* = 25), bolt-action rifles (*n* = 12), semi-automatic rifles (*n* = 14), and fully automatic rifles (*n* = 2). The average age at which EXP participants first used a firearm was 14 years (range 6 to 21 yr).

### Firearm hearing protection use survey

**FIGURE 4:**
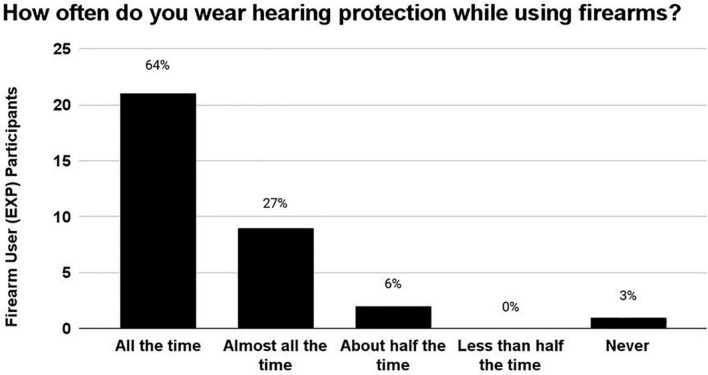
The number of occasions on which the EXP participants reportedly used firearms ranged from 5 to 200 occasions. During these occasions of firearm use, 91% of EXP participants reportedly wore hearing protection either “All of the time” or “Almost all the time”, indicating that this cohort used HPDs consistently.

After the EXP participants reported the estimated number of occasions on which they had used a firearm, they were asked to report how often they wore hearing protection devices (HPDs) during those occasions of use (Figure 4). 64% wore HPDs “all the time,” 27% wore HPDs “almost all of the time,” 6% wore HPDs “half of the time,” 0% HPDs “Less than half the time,” and 3% wore HPDs “never.” In sum, 91% of EXP participants (30/33) reported wearing HPDs either “all the time” or “almost all the time,” indicating that HPD use was consistent in this cohort of young-adult firearm users.

### Hearing protection device attenuation – Laboratory setting

**FIGURE 5:**
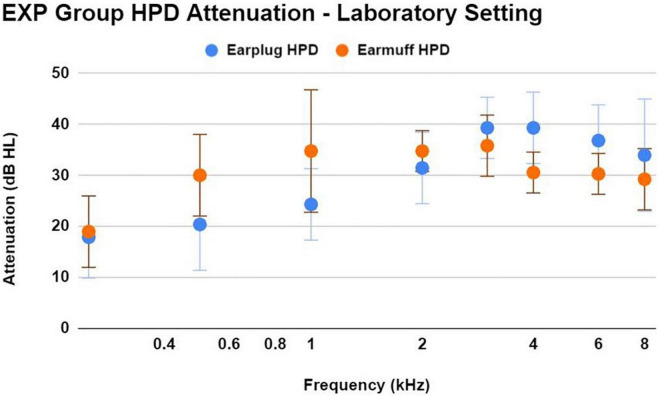
This figure shows the mean ± 1SD of HPD attenuation for an earplug-type HPD (3M EAR Classic Earplugs; 29 dB NRR) and an earmuff-type HPD (3M H10A Peltor Optime; 30 dB NRR), as demonstrated by EXP participants in a laboratory setting without any instruction regarding HPD fit. Participants were asked to use the type of HPD that most closely resembled their own personal HPD. Participants demonstrated effective HPD attenuation regardless of HPD type. While real-world attenuation may be different than lab-based attenuation, the effective attenuation observed here is consistent with the participant’s normal-hearing status.

During the laboratory real-ear-attenuation at threshold (REAT) testing of HPDs, 14/33 EXP participants chose the earplugs (3M EAR Classic Earplugs; 29 dB NRR), and 19/33 EXP participants chose the earmuffs (3M H10A Peltor Optime; 30 dB NRR). The one EXP participant who reported not using HPDs chose earplugs. HPD attenuation measurements obtained in a laboratory with standardized HPD equipment (in lieu of personal HPD equipment) are limited in accuracy; nevertheless, it is interesting to document consistently effective HPD attenuation (Figure 5) in this laboratory setting across EXP participants, who did not receive any HPD fitting guidance or instruction from research personnel.

### Auditory differences between control and experimental groups

In this dataset, 31/33 EXP participants reported exposure to rifles, shotguns, or both. As such, analysis of ear (right and left) was included as a repeated measure in the linear mixed model analyses, because firearm exposure is known to often cause asymmetrical auditory damage in the “off-handed ear” (the ear opposite the user’s dominant hand). The “off-handed” ear is more exposed to the blast when firing rifles or shotguns, but not when firing handguns (in which case, there is approximately equal sound exposure to both ears) (see Le et al., 2017 for review). All EXP participants in this study were right-handed. Therefore, if asymmetrical damage were to exist, greater deficits in the left ear than in the right ear would be predicted within the EXP group, but only in those participants who used rifles and/or shotguns (i.e., not solely handguns).

**FIGURE 6:**
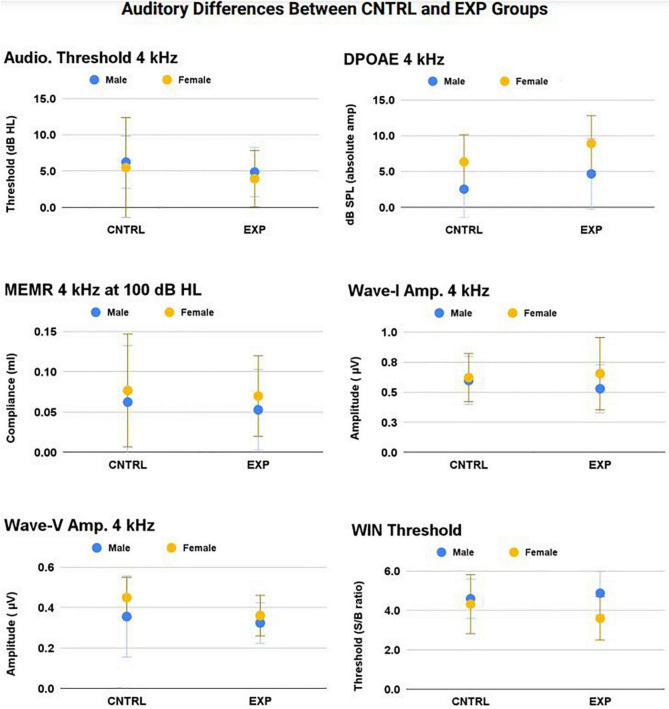
No statistically significant main effect nor interaction effect of firearm group was observed on audiologic outcomes (*p* > 0.05). The frequency at which noise-induced deficits were most likely to be observed between groups — 4 kHz — is shown here (mean ± 1SD) for audiometric threshold, DPOAE, MEMR, ABR Wave-I amplitude, ABR Wave-V amplitude, and WIN threshold (all *p’s* > 0.05).

Therefore, a series of linear mixed models with repeated measures (right and left ear) were used to test for the effects of ear, sex, noise history score, and group (EXP and CNTRL) on auditory measures. Group means ± 1SD for WIN threshold and all 4 kHz audiologic measurements (audiometry, DPOAE, MEMR, ABR) are shown in Figure 6 (right and left ear averaged), as 4 kHz was the frequency most likely to exhibit noise-induced deficit. The 4 kHz group data are representative of group differences (EXP and CNTRL) tested at all frequencies, and there were no statistically significant differences detected as a function of group.

#### Auditory brainstem response

In the Wave-I amplitude Click response, a main effect of sex was observed (*p* < 0.001), with female amplitude larger than male amplitude (female mean = 0.55 μv ± 0.18; male mean = 0.45 μv ± 0.16). In the Wave-1 amplitude 4 kHz response, a main effect of sex was observed (*p* = 0.002), with female amplitude larger than male amplitude (female mean = 0.63 μv ± 0.21; male mean = 0.56 μv ± 0.20). These significant sex differences are established in the literature (Jerger and Hall, 1980).

#### 4 kHz DPOAE response

**FIGURE 7:**
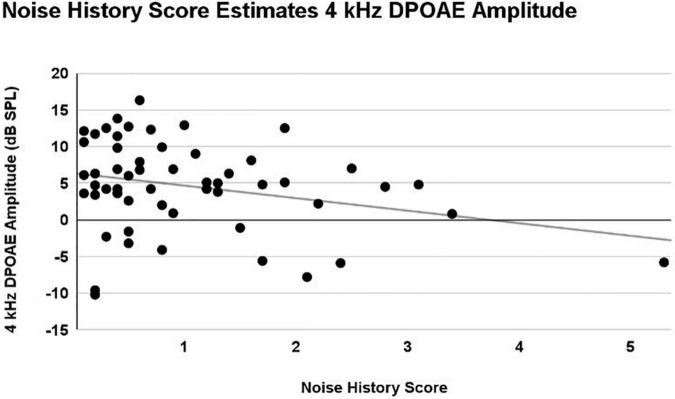
A linear mixed model with repeated measures (right and left ear) was used to analyze the effects of ear, sex, firearm group, and Noise History Score on measures of auditory function. Only an effect of Noise History Score was statistically significant (*p* = 0.03) in the 4 kHz DPOAE amplitude (dB SPL) measurement. Right and left ear averaged data are shown here, demonstrating a weak correlation, with 4 kHz DPOAE amplitude decreasing as Noise History Score increased (*R* = 0.28).

A main effect of Noise History Score on 4 kHz DPOAE amplitude was observed (p = 0.03), with DPOAE amplitude decreasing as Noise History Score increases. This main effect was statistically significant with and without inclusion of the single outlier data point (Noise Score = 5.3). Right and left ear DPOAE amplitudes were averaged and plotted in Figure 7. Pearson’s R = 0.28 including the Noise History Score outlier.

#### Auditory function in “High-risk” EXP participant subgroup

**FIGURE 8:**
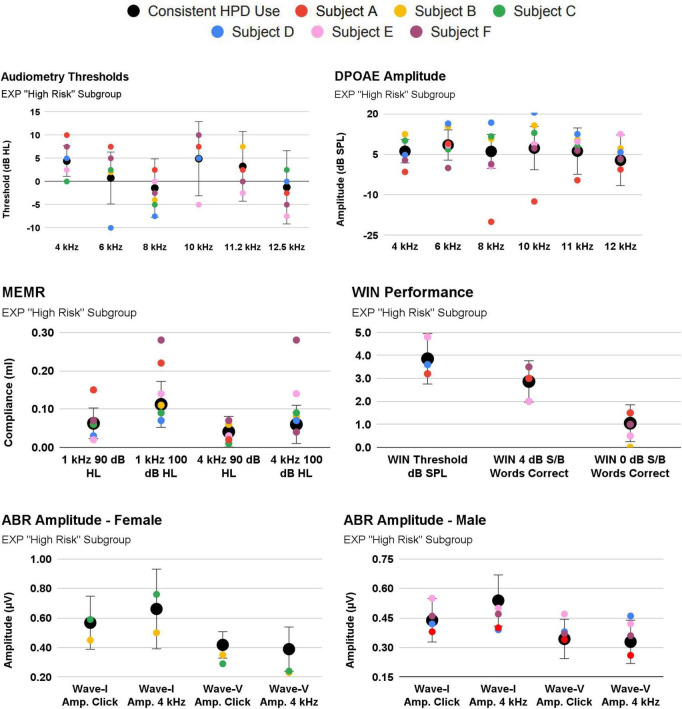
3/33 EXP participants reported inconsistent use of hearing protection [Subject (A–C)]. Their audiologic responses are plotted against the EXP group mean [EXP mean calculated after omitting Subjects (A–C)]. Also shown are Subjects (D–F), as these they reported the highest occasions of firearm use (albeit, while wearing consistent hearing protection).

Participants were assigned to a new, “High-Risk” EXP Participant subgroup if they (A) reported inconsistent HPD use, or (B) reported ≤50 occasions of firearm use. As noted above, 30 EXP participants reported consistent HPD use (either “all the time” or “most of the time”), while only three participants reported HPD use “about half the time” or “never”, precluding systematic analysis of a “consistent vs. inconsistent HPD user group”. Nevertheless, firearm use trends in this “High Risk” EXP subgroup are reported in Table 1. Auditory function trends within this subgroup are plotted in Figure 8.

**TABLE 1:** Unique firearm user subgroup: Inconsistent HPD use and highest occasions of use.

	HPD Consistency	Lifetime firearm use occasions	Sex
Subject A	Less than half the time	50	Male
Subject B	Less than half the time	5	Female
Subject C	Never	5	Female
Subject D	Almost all the time	200	Male
Subject E	All the time	100	Male
Subject F	All the time	200	Male

**Specifically, Subject A** (red symbols; Figure 8) exhibits an important relationship consistent with cochlear damage evidenced in youth firearm users in Laffoon et al. (2019). Subject A reported both inconsistent HPD *and* 50 occasions of firearm use. Despite a normal audiogram, Subject A tended to exhibit poorer cochlear function than that of the consistent HPD users. For example, Subject A fell outside 1SD from the mean in a “notch”-like audiometry pattern (4 kHz, 6 kHz), and their DPOAE function was outside 1SD at 4 kHz and 8-11.2 kHz. Interestingly, Subject A’s MEMR function was outside 1SD from the mean, but was better (stronger) than the mean. By contrast, Subject A’s auditory function was within 1SD on measures such as ABR and WIN. Subject A’s ABR function (while within 1SD of the mean) is regularly below the mean, yet the data indicate that the damage is not selectively neural, as audiometry and DPOAE function are also poorer than the mean.

## Discussion

Firearm noise exposure has been shown to be hazardous in multiple reports that include not only adult populations (military personnel, hunters, and recreational firearm users) (for review, see Sonstrom Malowski et al., 2022), but also youth firearm users (Laffoon et al., 2019). Importantly, some evidence is suggestive of a cochlear synaptopathy phenotype in Veterans with high noise exposure, as well as in civilian firearm users (Bramhall et al., 2017). Participants in the Bramhall et al. (2017) investigation were between the ages of 19–35 yr, exhibited normal hearing in at least one ear (32 participants met criteria in both ears; 34 met criteria in just one ear) and exhibited present OAEs but reduced ABR Wave-I amplitude relative to control groups. Building upon this work, the current investigation was designed to explore a more complete battery including MEMR and WIN function, a younger cohort (18–25 yr), and participants who met the ≤ 20 dB HL threshold criterion in both ears instead of a single ear. While deficits were anticipated in the current young-adult firearm user group, based on the cochlear deficits in youth firearm users documented by Laffoon et al. (2019) and the Wave-I deficits documented in Bramhall et al. (2017), neither cochlear nor neural deficits were observed in the group mean data.

One possible explanation is that synaptic recovery, as shown in guinea pigs, gerbils, and mice (Kauer et al., 2019; Hickman et al., 2020; Jeffers et al., 2021), may also occur in humans, thereby explaining the lack of hidden hearing loss pathology observed in the current investigation. Synapse recovery remains incompletely understood, with some data suggesting better recovery of high-SR fibers than low-SR fibers (see for example Jeffers et al., 2021).

However, the more logical explanation for the lack of auditory deficits in the current firearm user participants is that their consistent HPD use (91% reported use “all the time” or “almost all the time”) prevented cochlear and neural deficits akin to those reported by others. Further, EXP participants demonstrated appropriate HPD attenuation when self-fit for laboratory REAT testing. It is not so surprising that young-adult participants who met normal-hearing sensitivity inclusion criteria in both ears also overwhelmingly reported consistent use of HPDs. Hearing protection can effectively prevent TTS even after extreme types of noise exposure, such as firing 40 rounds from an automatic machine gun (Le Prell et al., 2011).

The results from this study highlight a critical issue for future research: if evidence of noise-induced cochlear synaptopathy is to be searched for within populations of firearm users, it is essential to understand the patterns of HPD use within the study population. The urgent need to consider HPD use as part of the study design is supported by the individual participant data, with Subject A (Figure 8), who reported inconsistent HPD use (“less than half the time”) and many occasions of past firearm use (reportedly, 50 occasions). Subject A exhibited poorer cochlear function than consistent HPD users, even though all participants had normal audiograms. It is not surprising that a firearm user who uses HPDs “less than half the time” exhibits cochlear pathology (inferred from the decreased DPOAE amplitudes). Because of the potential for combined cochlear and neural pathologies, Bramhall et al. (2022) recently used a computational model to re-evaluate the data from Bramhall et al. (2017) and predict synaptopathic loss based on both the measured ABR and DPOAE responses (Bramhall et al., 2022).

Although the data were initially analyzed for group differences based on firearm group (CNTRL and EXP), it was also of interest to analyze auditory differences based on Noise History Score. This was particularly true given that 91% of EXP participants reported wearing HPDs either “all the time” or “almost all the time” during firearm use, but only 4% of all participants used HPDs during non-firearm noise exposures. It is crucial to note that, in this dataset of normal-hearing, young participants, the DPOAE 4 kHz amplitude was negatively correlated with the participant’s noise exposure history score. Per the definition of a selective, noise-induced synaptopathic loss, cochlear function must be fully intact. Exploring patterns of function, rather than clinically binary categorization of “normal” and “abnormal” function, is encouraged when analyzing cochlear deficits in participants with “clinically normal” audiograms.

In summary, young firearm users seemed likely to be a population that could experience very large TTS, similar to the degree of TTS that has been necessary to induce selective synaptopathy across animal models. However, firearm exposure (especially with inconsistent or improper HPD use) seems much more likely to result in overt, cochlear hearing loss, rather than a highly selective hidden hearing loss. Theoretically, a very specific population of firearm users may exist who have experienced acute (or repeated), severe TTS after firearm exposure, followed by complete recovery of bilateral audiometric thresholds and otoacoustic emissions, despite persistent synaptic damage. However, in consideration of (1) the preserved auditory health observed in the current cohort of firearm users with consistent HPD use, and (2) the multitude of studies showing cochlear deficit in firearm users with inconsistent or improper HPD use, we believe that selective neural injury in the absence of cochlear injury would be the exception and not the rule. At this time, prevention of overt (not hidden), cochlear damage remains the leading auditory concern for firearm users and non-firearm users.

Although young, firearm users with normal-hearing sensitivity seemed likely to be at risk for noise-induced cochlear synaptopathy based on Bramhall et al. (2017) and Laffoon et al. (2019), the participants in this study did not exhibit any deficits suggestive of a “hidden hearing loss” driven by noise-induced cochlear synaptopathy. It seems most likely that the EXP group effectively attenuated firearm noise exposure via consistent HPD use, and therefore failed to develop permanent cochlear or neural damage exceeding that of the control population, who had never been exposed to firearm noise. Hearing protection is routinely recommended for firearm users (e.g., Lobarinas et al., 2016; Meinke et al., 2017), including youth target shooters (Meinke et al., 2014). As such, it is encouraging to document that in this cohort, hearing loss has thus far been prevented in firearm users who report consistent HPD use.

## Summary and conclusion

The purpose of this study was to recruit and retrospectively analyze a human cohort we believed would be likely to exhibit evidence of noise-induced cochlear synaptopathy; normal-hearing, young-adult firearm users. This investigation differed from previous studies with similar goals and cohorts in that all participants were young (≤ 25 yr), exhibited bilaterally normal-hearing (thresholds 0.25–8 kHz ≤ 20 dB HL), and included a full battery of audiometry, extended high-frequency audiometry, DPOAE, MEMR, ABR, and WIN.

70 participants were enrolled in this study and were separated into either the Firearm User group (EXP) (5 + occasions of firearm use in their lifetime) or the Non-Firearm User group (CNTRL) (0 occasions of firearm use in their lifetime). Consistent hearing protection use, reported across 91% of EXP participants, may explain why no measure of auditory performance significantly differed between the CNTRL and EXP groups. Consistent hearing protection use may also explain why no significant left ear auditory deficit asymmetries were observed within the entirely right-handed EXP group. One firearm user participant, categorized as “high risk” due to their inconsistent use of hearing protection during many occasions of firearm use, exhibited significantly worse cochlear function (despite clinically normal audiometry thresholds) than those who used hearing protection consistently. Across all participants (CNTRL and EXP), increased exposure to non-firearm sources of noise was correlated with decreased 4 kHz DPOAE amplitude. Taken together, no evidence of “hidden hearing loss” was found in this cohort; overt (not hidden), cochlear damage remains the primary threat to the auditory system in human populations that experience acute or repeated severe TTS.

## Data availability statement

The raw data supporting the conclusions of this article will be made available by the authors, without undue reservation.

## Ethics statement

This study was approved by the Institutional Review Board at the University of Texas at Dallas. Written informed consent was obtained from participants prior to study enrollment. Participants were recruited from the University of Texas at Dallas campus in Richardson, Texas, and the Callier Center for Communication Disorders in Dallas, Texas. A digital flyer including study information was also shared via undergraduate and graduate student social media networks. The digital flyer contained a website link to a Qualtrics survey (a licensed, online survey software) which automatically screened interested participants to determine if they met the inclusion criteria. All study procedures were performed using dedicated clinical research equipment located at the Callier Center for Communication Disorders in Dallas, TX. All study procedures were conducted by a licensed audiologist or graduate students in the Doctor of Audiology program. Participants were allowed to withdraw from the study at any time, and they were compensated for their laboratory visit.

## Author contributions

SG contributed to the study design, data collection, data interpretation, and writing the manuscript. CL contributed to the study design, data interpretation, and writing the manuscript. Both authors contributed to the article and approved the submitted version.

## Footnotes

1
https://knowyournoise.nal.gov.au/

## Abbreviations

ABR: auditory brainstem response
ASSR: Auditory Steady State Response
dB: decibel
dBA: A-weighted decibel
dB HL: decibel hearing level
dB S/B: decibel signal to babble ratio
dB SPL: decibel sound pressure level
DPOAE: distortion product otoacoustic emission
ECochG: Electrocochleography
EHF: extended high frequency
HPD: hearing protection device
Hz: hertz
IHC: inner hair cell
kHz: kilohertz
NIOSH: National Institute on Occupational Safety and Health
NU-6: Northwestern University Auditory Test Number 6
OHC: outer hair cell
OSHA: Occupational Safety and Health Administration
PTS: permanent threshold shift
SRT: Speech Recognition Threshold
TTS: temporary threshold shift
WRS: Word Recognition Score
WIN: Words-in-Noise.
